# Impacts of phosphate-solubilizing bacterium strain MWP-1 on vegetation growth, soil characteristics, and microbial communities in the Muli coal mining area, China

**DOI:** 10.3389/fmicb.2024.1500070

**Published:** 2024-12-05

**Authors:** Yanru Wu, Wenquan Yang, Jiancun Kou, Qinyao Li, Jiaqing Liu, Lu Chi, Yangcan Zhang, Qian Liu, Yanghua Yu

**Affiliations:** ^1^College of Grassland Agriculture, Northwest A&F University, Yangling, China; ^2^Key Laboratory of the Alpine Grassland Ecology in the Three Rivers Region (Qinghai University), Ministry of Education, Xining, China; ^3^College of Life Sciences, Northwest A&F University, Yangling, China

**Keywords:** phosphate solubilizing bacterium, vegetation growth, soil microbial communities, soil nutrient, high-altitude mining area

## Abstract

Due to the cold climate and low soil nutrient content, high-altitude mining areas are challenging to restore ecologically. Their poor nutrient content may be ameliorated by introducing specific microorganisms into the soil. This study aims to evaluate the effects of a highly efficient phosphate solubilizing bacterium MWP-1, *Pseudomonas poae*, on plant growth, soil nutrients in remedying the soil of the high-altitude Muli mining area in Qinghai Province, and analyze its impact on microbial communities through high-throughput sequencing soil microbial communities. The results showed that MWP-1 significantly increased the content of soil available phosphorus by >50%, soil organic matter and total nitrogen by >10%, and significantly increased the height, coverage, and aboveground biomass of vegetation by >40% in comparison with the control (*p* < 0.05). MWP-1 mainly affected the composition of the soil bacterial communities at the taxonomic level below the phylum. Its impact on soil fungal communities occurred at the phylum and below taxonomic levels. In addition, MWP-1 also significantly improved the diversity of soil bacterial and fungal communities (*p <* 0.05), and changed their functions. It also significantly altered the relative abundance of genes regulating phosphorus absorption and transport, inorganic phosphorus dissolution and organic phosphorus mineralization in the bacterial community (*p <* 0.05). It caused a significant increase in the relative abundance of the genes regulating nitrogen fixation and nitrification in nitrogen cycling (*p <* 0.05), but a significant decrease in the genes regulating phospholipase (*p <* 0.05). Although sequencing results indicated that *Pseudomonas poae* did not become the dominant species, its dissolved phosphorus elements can promote plant growth and development, enrich soil nutrient content, and affect the succession of microbial communities, enhance ecosystem stability, with an overall positive effect on soil remediation in the mining area.

## 1 Introduction

The Muli coalfield in Qinghai Province of Northwest China is an important source of coking coal (Wang, 2010), where mining has caused serious impacts on the eco-environment (Hou et al., 2019). Successful restoration of the mining-damaged ecosystem in this area is difficult due to excessive excavation, slag accumulation (Liu and Liu, 2020), lack of soil nutrients, and its high altitude and cold climate. Of all mineral nutrients, phosphorus is the most important element to plant growth and development second only to nitrogen. Phosphorus deficiency limits plant growth and reduces dry plant weight by approximately 0.2% (Rawat et al., 2021). Besides, most of the phosphorus in the soil is bound to cations and hence difficult for plants to absorb and utilize (Abbaszadeh-Dahaji et al., 2020). Soil phosphorus deficiency can be effectively remedied via applying phosphate fertilizer during ecological restoration in the Muli mining area (Nussbaumer et al., 2016). However, the phosphorus fertilizer applied to the soil is easily fixed by metal ions to form insoluble phosphate, which reduces fertilizer efficiency (Ma et al., 2021). Additionally, the Muli mining area is located in the source area of the Datong River, an important tributary of the Yellow River. The extensive use of phosphate fertilizers can easily cause water pollution and degrade water quality in downstream areas (Barrow, 2015). The alternative is to make use of microbial fertilizers such as phosphate solubilizing bacteria (PSB) that may serve as a bridge for regulating damaged vegetation, soil, and microorganisms in high-altitude mining areas, and are a green and efficient ecological restoration material (González et al., 2019).

As a type of soil microorganisms, PSB can convert organic phosphorus into inorganic phosphorus or inorganic phosphorus into soluble phosphorus (Timofeeva et al., 2022). They can promote plant growth by synthesizing plant hormones (Khan et al., 2017; Kudoyarova et al., 2017) and increasing the absorption of trace elements such as zinc and iron (Al-Enazy et al., 2017). Furthermore, PSB can also alleviate the lack of available phosphorus in soil, improve soil microbial communities (Pan and Cai, 2023), prevent soil compaction, and reduce environmental pollution (Canbolat et al., 2006). So far, researchers have shown that PSB can increase phosphorus uptake and improve plant yield in both indoor pot and field experiments (Rosas et al., 2006; Zheng et al., 2019; Alam et al., 2022). However, there is a lack of understanding about their effects on soil nutrients and soil microbial communities in high-altitude mining areas. Due to their harsh environment and significantly different microbial communities from those of low-altitude areas, it is difficult to replicate the expected results using exogenous rhizosphere growth promoting bacteria (Bashan et al., 2014). The mining areas may face the risk of low microbial activity that reduces the growth-promoting effects of fertilizers, and causes the invasion of exogenous microorganisms harmful to the original microbial communities (Gu et al., 2021). The use of growth-promoting bacteria suitable for soil remediation in high-altitude regions should be able to adapt to the local climate without causing negative impacts on the local microbial communities (Li et al., 2022). Therefore, it is significant to explore PSB capabilities in the high-altitude region and understand their possible impacts on the soil microbial communities, and assess their potential as an effective measure to facilitate vegetation restoration in high-altitude mining areas.

In this study, highly efficient PSB isolated from high-altitude regions were used in the ecological restoration of the Muli mining area, with three main objectives of study: (1) to determine the effects of PSB on vegetation growth in high-altitude mining areas; (2) to assess the effects of PSB on soil chemical properties in high-altitude mining areas, and (3) to explore the effects of PSB on bacterial and fungal communities, as well as the functional characteristics of bacterial and fungal communities related to soil nitrogen and phosphorus cycling in high-altitude mining areas.

## 2 Materials and methods

### 2.1 Overview of the experimental site

The experimental site is located in the Jiangcang mining area of the Muli coalfield in Gangcha County, Qinghai Province, northeast of the Qinghai Tibet Plateau in China (38°02′-38°03′N, 99°27′-99°35′E). Its altitude ranges from 3,800 to 4,200 m. The annual temperature ranges from the minimum of −36°C to the maximum of 19.8°C, with an average of −5.1 ~ −4.2°C. Annual precipitation averages 500 mm, about one third of the annual evaporation (1,418 mm). Precipitation is mainly concentrated from July to September, while snowfall is concentrated from January to May (Cao et al., 2010). The experimental site is located in the No. 5 mining area of Jiangcang. In 2016, the slag mountain was unloaded through slope flattening and mechanical leveling. Large rocks, gravel, and coal gangue were removed to form a soil substrate about 30 cm thick, on which artificial grassland was established. The sowed grass species included *Elymus breviaristatus* “Tongde,” *Puccinelia tenuiflora* “Tongde,” *Festuca sinensis* “Qinghai,” *Poa cryophila* “Qinghai,” and *P. pratensis* “Qinghai.” The sowing dosage of grass seeds totaled 22.5 g/m^2^, with an equal mass ratio among the five species. The seeds were mixed with grass specific fertilizer (NPK compound fertilizer, total nutrients ≥35%, N 18%, P_2_O_5_ 12%, K_2_O 5%) and manually sown. The dosage of grass specific fertilizer was set at 25.5 g/m^2^. Then, the surface of the sowed fields was gently raked and leveled to bury the seeds, and finally covered with non-woven fabric for insulation and germination promotion (Li et al., 2019). Afterwards, the site was left idle for 6 years (e.g., no fertilization, grazing or other treatments), beyond which the established artificial grassland started to degrade even severely.

### 2.2 Test materials

The tested bacterial agent, PSB strain MWP-1 (*Pseudomonas poae*), was secured through screening the alpine meadow soil. This strain can grow normally at a low temperature of 10°C, with a phosphorus release rate of 14.61%. It can produce diverse organic acids such as oxalic acid, lactic acid, acetic acid, citric acid, and succinic acid, with a total organic acid content of 7461.9 μg/mL. Besides, it can also produce indole-3-acetic acid (IAA), ACC deaminase, and iron carriers, and has shown a good promoting effect in indoor pot experiments.

### 2.3 Experiment design

Two plots (3 m × 3 m) were randomly selected at the artificially sowed grassland, one treated with PSB and the other untreated as the control. The PSB strain MWP-1 was propagated and cultured using LB liquid medium, with OD_600_ adjusted to 1. After dilution by 100 times, it was evenly sprayed over the bare plots at a dosage of 1 L/m^2^ in June 2022 when the artificial vegetation greened. The control plot was sprayed by an equal amount of sterile water. Vegetation in the two plots was surveyed and soil sampled at the end of the growing season in August.

### 2.4 Vegetation survey and soil sample collection

Three sub-plots (1 m × 1 m) (i.e., three replicates) were randomly selected in each plot, within which vegetation coverage was measured using the needle puncture method, and 20 replicates were randomly selected to measure plant height. After uniform cutting, the biomass within each subplot was measured. In the same subplot, soil samples were collected at a depth of 0–20 cm using a soil drill (inner diameter = 5 m) and the multi-point mixing method. During sampling sterile gloves and sterile self-sealing bags were used to avoid contamination. Ten soil samples were collected from each subplot. After the removal of plant roots and stones in the soil samples, the 10 samples from the same subplot were mixed thoroughly to form one composite sample, from which ~2 g of soil were taken and transferred into a cryovial at 6 replicates. The cryovials were kept in dry ice for sequencing and analysis of the microbial communities. The remaining soil samples were naturally air-dried in the laboratory and then tested for soil physicochemical properties.

### 2.5 Determination of soil nutrients

Several soil nutrients were analyzed in this study, including soil organic matter (SOM), total nitrogen (TN), total phosphorus (TP), total potassium (TK), nitrate, and available potassium (AK), of which SOM was determined by the K_2_Cr_2_O_7_-H_2_SO_4_ oxidation method. Soil TN was determined using the H_2_SO_4_ de-boiling-distillation method. Soil TP was determined by spectrophotometry after HClO_4_-H_2_SO_4_ de-boiling-and-boiling. Soil TK was determined by flame photometry. Soil nitrate and ammonium nitrogen were determined by 1 mol/L KCl extraction using a continuous flow injection analyzer (NO_3_^−^-N, NH_4_^+^-N). After NaHCO_3_ extraction, available phosphorus (AP) was determined by spectrophotometry. After NH_4_OAc extraction, soil available potassium (AK) was determined by flame photometry. Specific determination steps could be found in *Soil Analysis Methods* (Sparks et al., 1996).

### 2.6 DNA extraction and 16S rRNA, ITS rRNA sequencing of soil microorganisms

Total genomic DNA samples were extracted using the OMEGA Soil DNA Kit (M5635-02) (Omega Bio-Tek, Norcross, GA, United States), and stored at −20°C prior to further analysis. The quantity and quality of extracted DNAs were measured using a Nanodrop NC2000 spectrophotometer (Thermo Fisher Scientific, Waltham, MA, United States) and agarose gel electrophoresis, respectively. PCR amplification of the V3-V4 region of soil bacterial 16S rRNA genes was performed using forward primer 338F (5′-ACTCCTACGGGAGGCAGCA-3′) and reverse primer 806R (5′-GGACTACHVGGGTWTCTAAT-3′). PCR amplification of the V1 region of soil fungal ITS rRNA gene was performed using forward primer 1737F (5′-GGAAGTAAAAGTCGTAACAAGG-3′) and reverse primer 2043R (5′-GCTGCGTTCTTCATCGATGC-3′). Sample-specific 7-bp barcodes were incorporated into the primers for multiplex sequencing. The PCR components contained 5 μL of buffer (5×), 0.25 μL of Fast pfu DNA Polymerase (5 U/μL), 2 μL (2.5 mM) of dNTPs, 1 μL (10 uM) of each Forward and Reverse primer, 1 μL of DNA Template, and 14.75 μL of ddH_2_O. Thermal cycling consisted of initial denaturation at 98°C for 5 min, followed by 25 cycles consisting of denaturation at 98°C for 30 s, annealing at 53°C for 30 s, and extension at 72°C for 45 s, with a final extension of 5 min at 72°C. PCR amplicons were purified with Vazyme VAHTSTM DNA Clean Beads (Vazyme, Nanjing, China) and quantified using the Quant-iT PicoGreen dsDNA Assay Kit (Invitrogen, Carlsbad, CA, United States). After the individual quantification step, amplicons were pooled in equal amounts, and pair-end 2 × 250 bp sequencing was performed using the Illlumina NovaSeq platform with NovaSeq 6000 SP Reagent Kit (500 cycles) at Shanghai Personal Biotechnology Co., Ltd. (Shanghai, China).

### 2.7 Sequence analysis

Microbiome bioinformatics were acquired with QIIME2 2019.4 in several steps (Bolyen et al., 2019). Briefly, raw sequence data were demultiplexed using the Demux plugin followed by primers cutting with Cutadapt plugin (Martin, 2011). Sequences were then quality filtered, denoised, and merged. And chimera was removed using the DADA2 plugin (Callahan et al., 2016). Non-singleton amplicon sequence variants (ASVs) were aligned with mafft (Katoh et al., 2002) and used to construct a phylogeny with fasttree2 (Price et al., 2009). After denoising all libraries, ASVs feature sequences and ASV tables were merged, with singletons ASVs removed. Taxonomy was assigned to ASVs using the classify-sklearn naïve Bayes taxonomy classifier in feature-classifier plugin (Bokulich et al., 2018) against the SILVA Release 132/UNITE Release 8.0 Database (Kõljalg et al., 2013).

Sequence data analysis was mainly conducted using QIIME2 and R packets (v3.2.0). ASV-level alpha diversity indexes, such as the Chao1 richness estimator, Shannon diversity index, and Pielou’s evenness, were calculated using the ASV table in QIIME2 and visualized as box plots. Beta diversity analysis was performed to investigate the structural variation of microbial communities across samples using Bray-Curtis metrics (Bray and Curtis, 1957) and visualized via principal coordinate analysis (PCoA). ASV-level ranked abundance curves were generated to compare the richness and evenness of ASVs among samples. A Venn diagram was generated to visualize the shared and unique ASVs among samples or groups using the R package “Venn Diagram,” based on the occurrence of ASVs across groups (Zaura et al., 2009). Microbial functions were predicted by PICRUSt2 (Phylogenetic investigation of communities by reconstruction of unobserved states) (Douglas et al., 2020) upon MetaCyc^1^ and KEGG^2^ databases. Based on the KEGG database, bacterial community function prediction analysis was conducted on the MWP-1-treated soil. After obtaining abundance data of metabolic pathways, PCoA was used to study the (dis)similarity of bacterial community functionality between MWP-1-treated and the control soil. And based on the MetaCyc database, fungal community function prediction analysis was conducted on soil in the study area treated with MWP-1. After obtaining abundance data of metabolic pathways, PCoA was used to study the differences in fungal community functionality between the MWP-1 treatment and the control. The KEGG database was searched for genes related to soil microbial nitrogen cycling (Supplementary Table S1) and phosphorus transformation (Supplementary Table S2) in different treatments. Redundancy analysis (RDA) was performed in the dplyr package of R.

## 3 Results and analysis

### 3.1 Effects of MWP-1 on vegetation and soil nutrients

**Figure 1:**
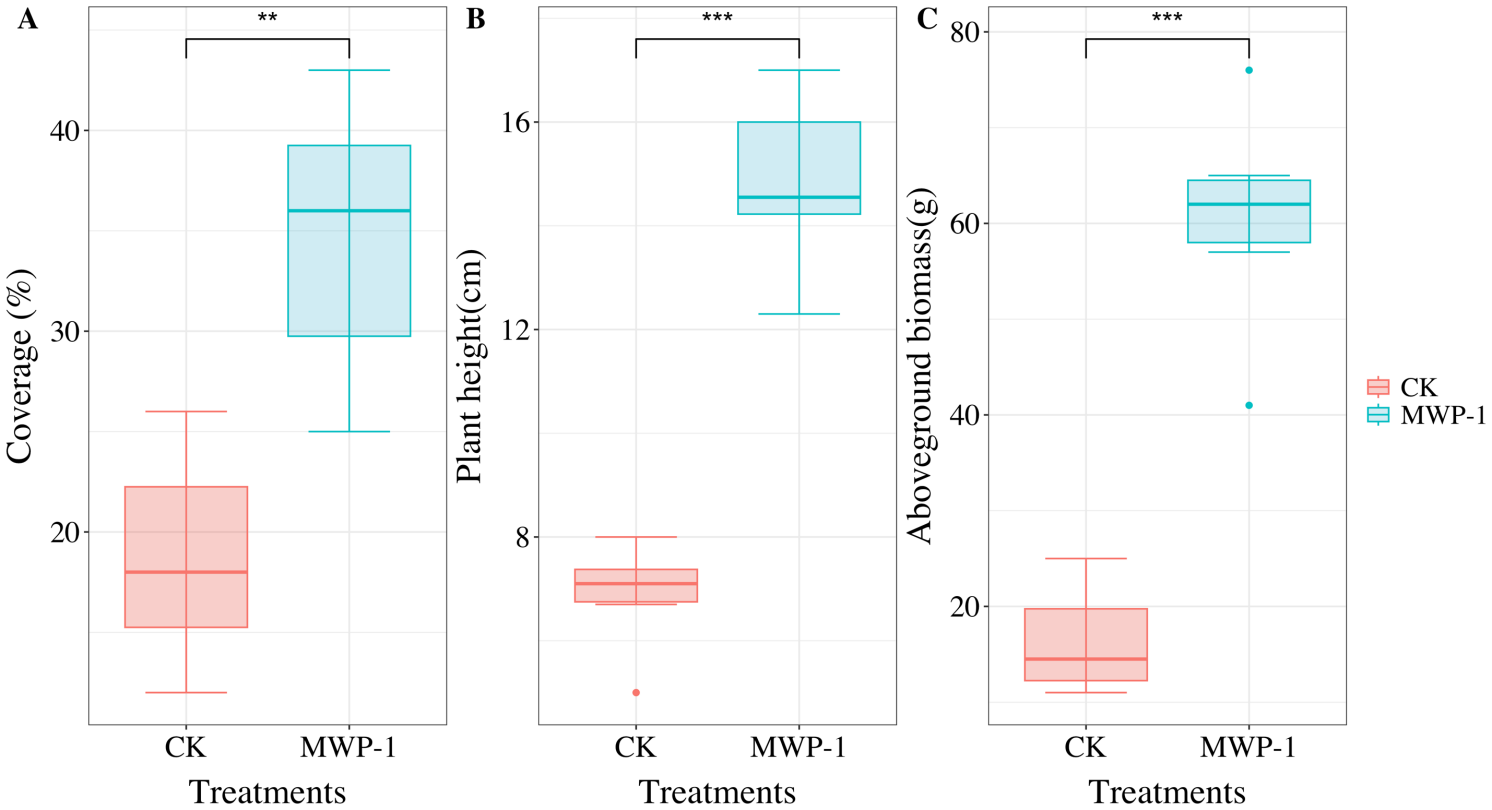
Coverage **(A)**, plant height **(B)** and aboveground biomass **(C)** of artificially seeded vegetation in the high-altitude mining area after applying strain MWP-1. **p < 0.01, ***p < 0.001.

**Figure 2:**
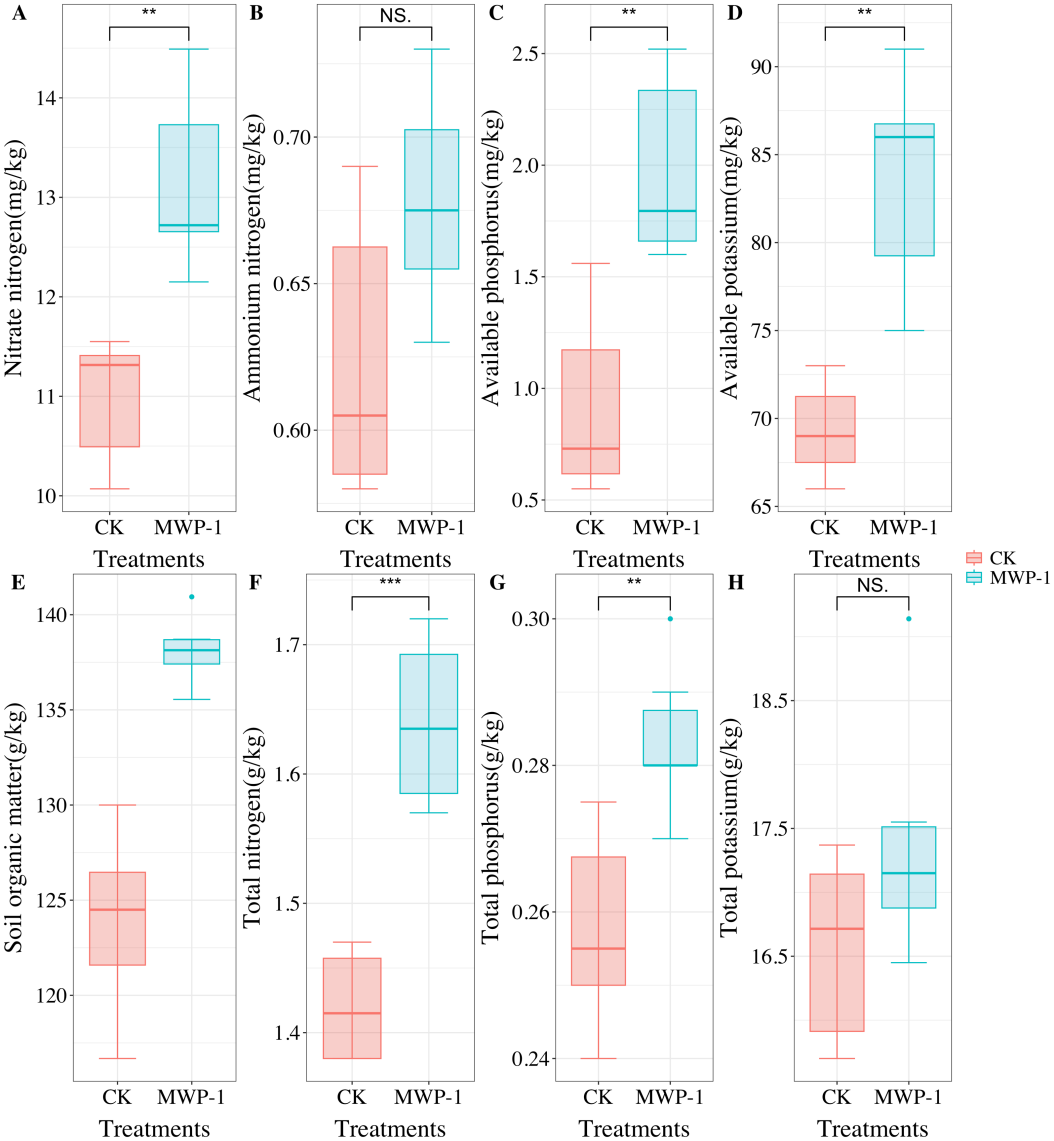
Nitrate nitrogen **(A)**, ammonium nitrogen **(B)**, available phosphorus **(C)**, available potassium **(D)**, soil organic matter **(E)**, total nitrogen **(F)**, total phosphorus **(G)** and total potassium **(H)** in the high-altitude mining area after the application of strain MWP-1. **p < 0.01, ***p < 0.001.

Vegetation had severely degraded from the sixth year of artificial seeding in the high-altitude mining area. However, after applying MWP-1, the coverage, height, and aboveground biomass of the artificially seeded vegetation significantly increased, (*p <* 0.05) in comparison with the control (Figure 1). Compared with the control, the soil samples treated with MWP-1 showed a highly significant increase in the contents of AP (Figure 2C) and total content (Figure 2G) (*p <* 0.01). As for nitrogen, both the TN content (Figure 2F), nitrate nitrogen content (Figure 2A), and AP (Figure 2D) in the soil significantly increased (*p <* 0.01), while the ammonium nitrogen content (Figure 2B) and TP (Figure 2H) did not show significant changes. The organic matter content in the soil also significantly increased (Figure 2E).

### 3.2 Effects of MWP-1 on soil bacterial communities

#### 3.2.1 Analysis of sequencing

**Figure 3:**
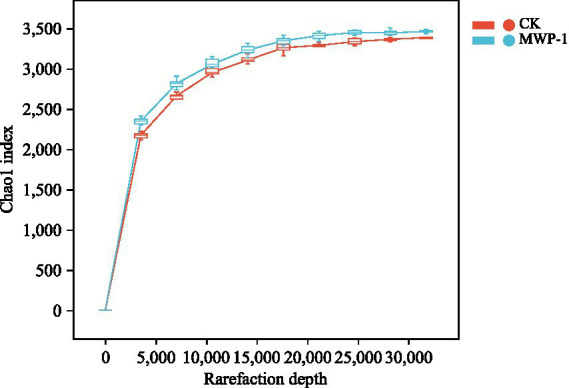
Sparse curve of sequencing sample dilution curve based on Chao1 index of the bacterial community.

The treatment and control samples were clustered to identify sequences and ASVs based on 100% similarity Sequence length distribution statistics performed on the bacterial communities indicated that an average of 71,397 valid sequences was measured per sample. After removing ASVs with a total sequence count of 1, a total of 53,333 sequences remained. The dilution curves (Figure 3) of the samples tended to flatten, indicating that the sequencing data were close to saturation and the sequencing depth was reasonable.

#### 3.2.2 Composition of the soil bacterial community

**Figure 4:**
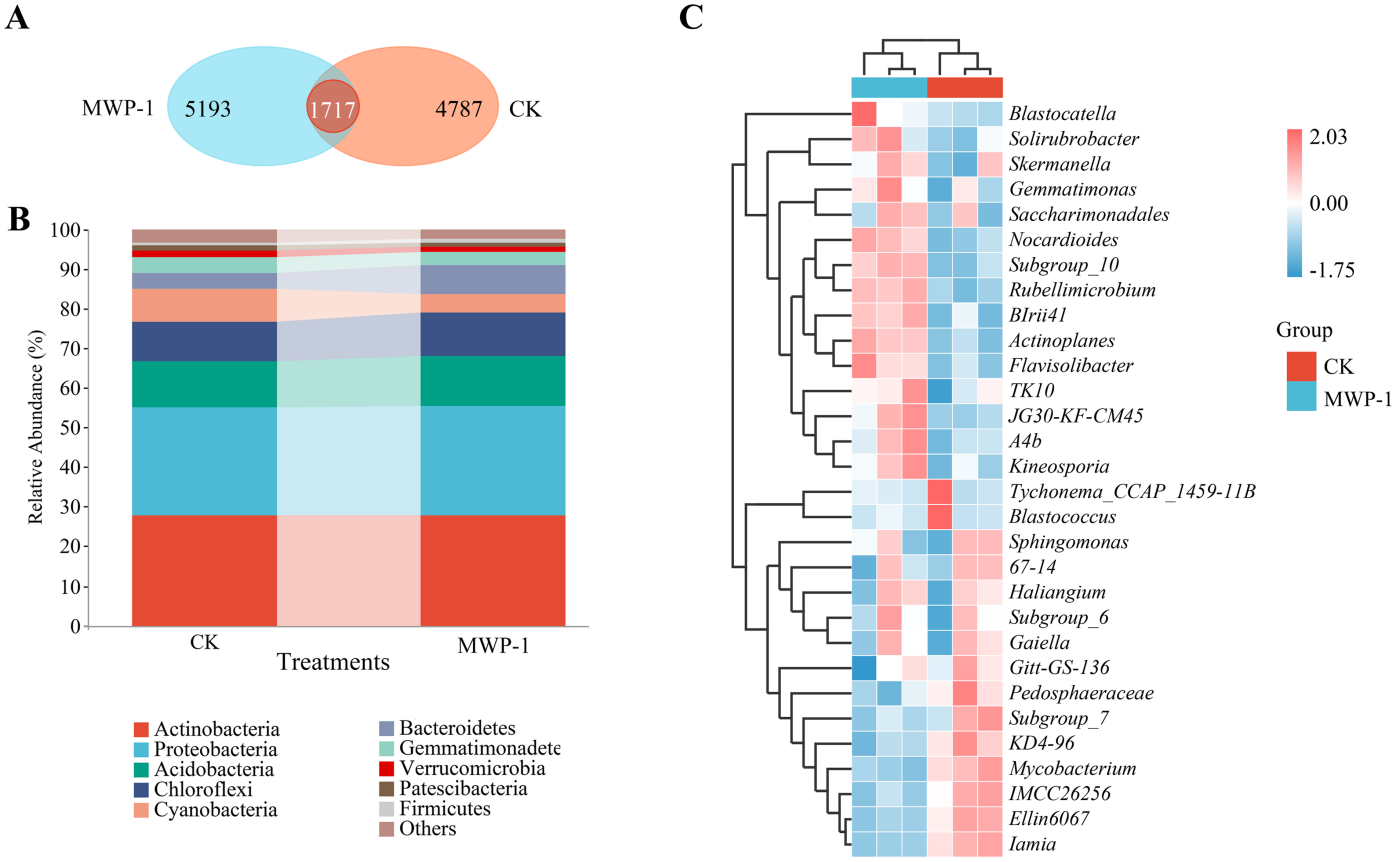
ASVs Wayne diagram **(A)**, horizontal community bar chart of bacterial phylum **(B)**, and thermal map of horizontal community composition of bacterial genus **(C)** in the high-altitude mining area after the application of strain MWP-1.

According to the Venn diagram (Figure 4A), a total of 11,697 ASVs were obtained from the treatment and the control after clustering, classification, and denoising. Some of them were merged to form a total of 1,717 ASVs, of which 4,787 ASVs were unique to the control, and 5,193 ASVs unique to the treatment. MWP-1 affected the number of ASVs in the soil of the high-altitude mining area.

Figure 4B showed the species composition of soil bacteria at the phylum level in the treatment. The main bacterial communities in the soil were Actinobacteria (27.7%), Proteobacteria (27.65%), Acidobacteria (12.59%), Chloroflexi (10.99%), Cyanobacteria (4.64%), and Bacteroidetes (7.52%) respectively. Verrucomibia (1.2%), Patescibacteria (1.2%), and Firmicutes (0.7%) were also detected, but their abundance was relatively low at only 0.1–5%.

Heatmap clustering revealed the community composition of different genera in the treatment (Figure 4C). The relative abundance of *Blastocatella, Solirubrobacter, Skermanella, Gemmatimonas, Saccharimondales, Nocardioids, Subgroup-10, Rubellimicrobium, BIrii41, Actinopales, Flavisolibacter, TK10, JG30-KF-CM45, A4b*, and *Kineosporia* was significantly enriched in the treatment, where the relative abundance of *Tychonema CCAP 1459-11B, Blastococcus, Pedosphaeraceae, Subgroup-7, KD4-96, Mycoplasma, IMCC26256, E11in6067*, and *Lamia* was significantly enriched in the control. Analysis of species composition at different levels of bacterial communities showed that MWP-1 mainly impacted the soil bacterial communities at the taxonomic level below the phylum.

**Figure 5:**
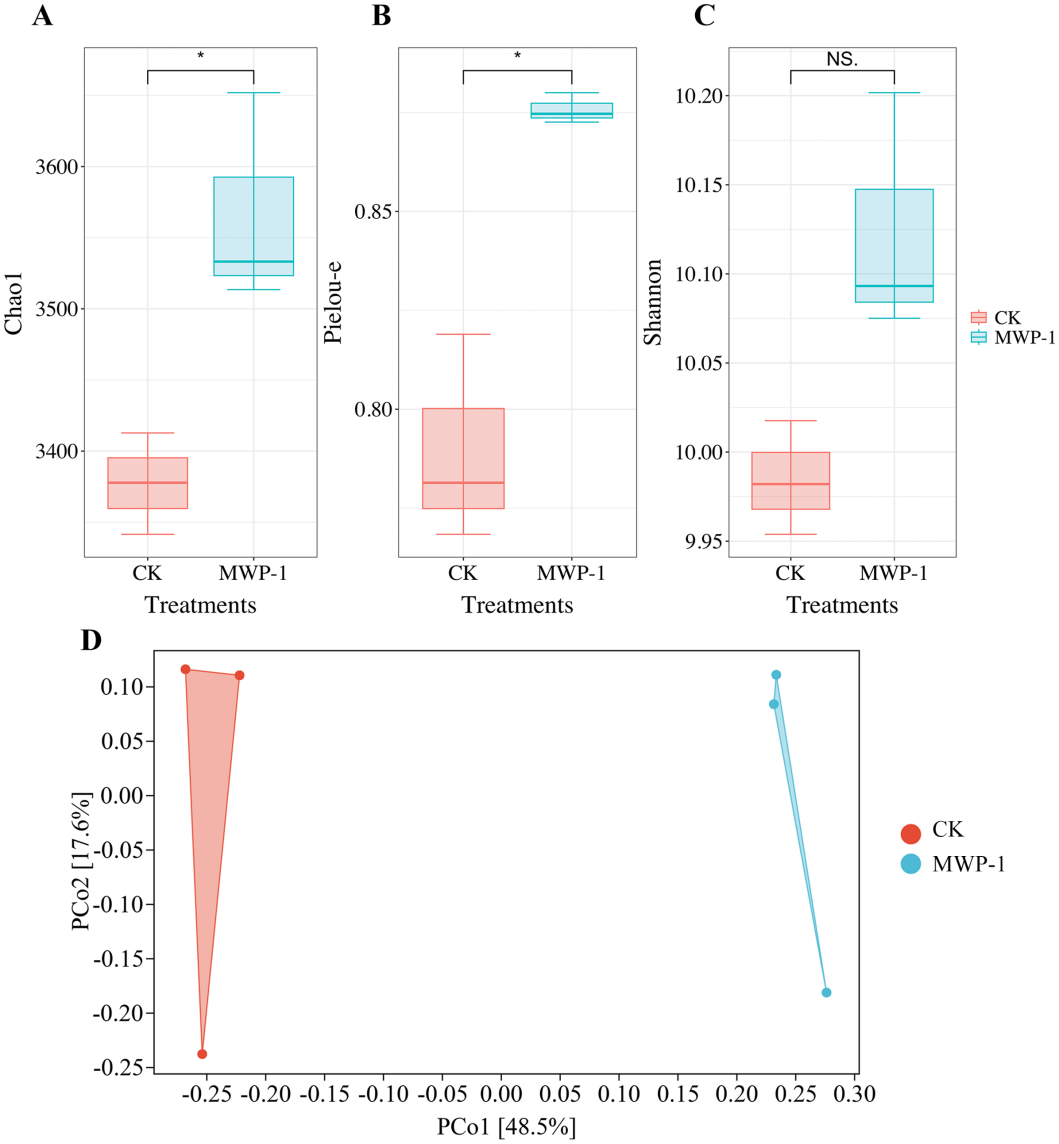
Alpha diversity indices of the high-altitude mining soil: Chao1 index **(A)**, Pielou-e index **(B)**, Shannon index **(C)** and beta diversity **(D)** of soil bacterial community. *p < 0.05.

The soil microbial alpha diversity analyzed included community richness measured by the Chao1 index, community diversity represented by the Shannon index, and community evenness represented by the Pielou-e index. The Chao1 index (Figure 5A) and Pielou-e index (Figure 5B) of the treatment significantly increased (*p <* 0.05), indicating that MWP-1 increased the richness and evenness of the bacterial communities in the soil. Bacterial community diversity showed an increasing trend, but the changes were not significant (Figure 5C). The beta diversity index from the bacterial community analysis based on the Bray-Curtis metrics between samples showed that PCo1 and PCo2 contributed 48.5 and 17.6% to the total variation, respectively (Figure 5D). The bacterial community of the soil samples treated with or without MWP-1 was significantly separated on the PCo1 axis of PCoA, indicating its impact on the soil bacterial community.

#### 3.2.3 Prediction of soil bacterial community functionality

**Figure 6:**
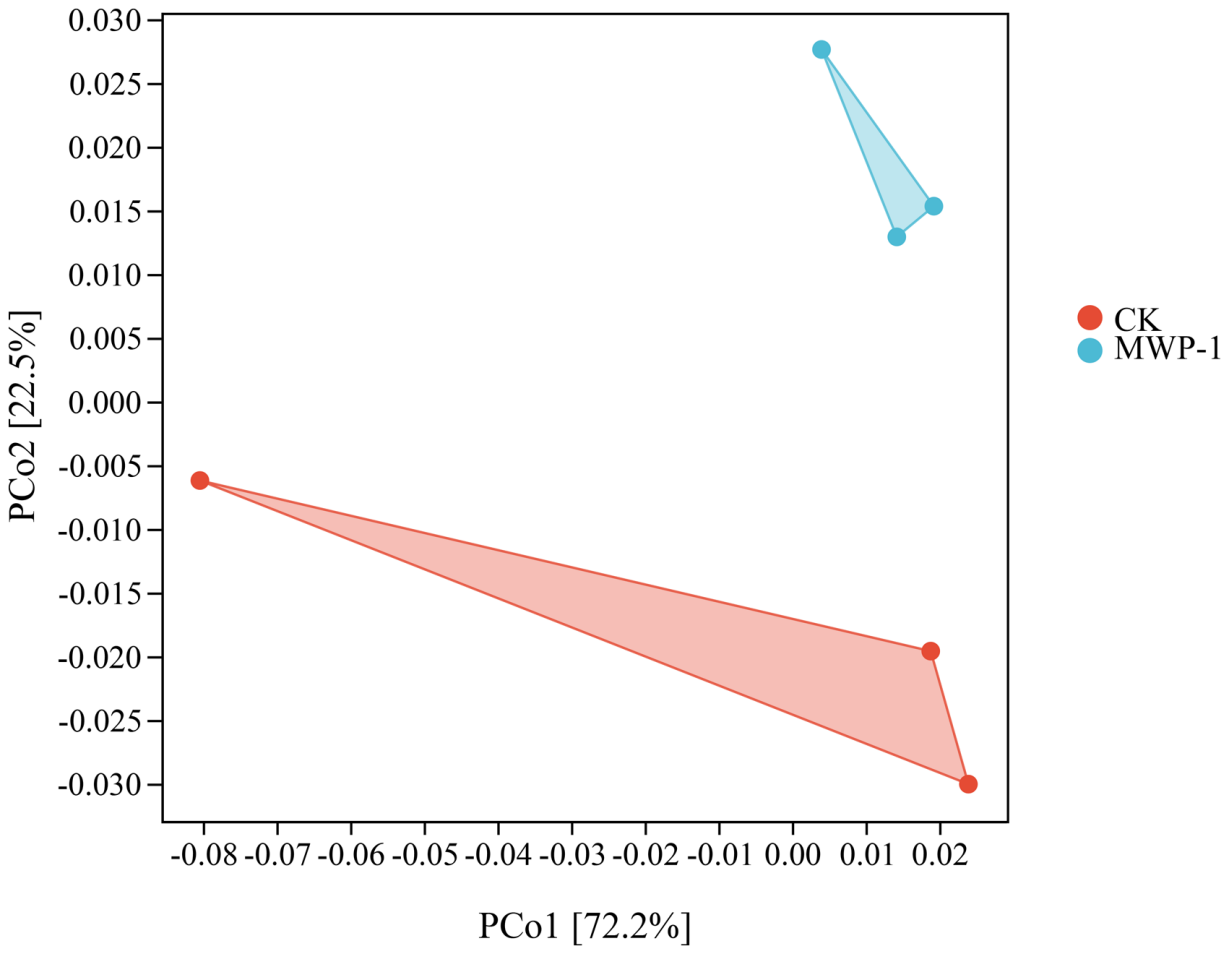
PCoA analysis outcome of soil bacterial community functional units.

The two latitudes of PCoA in the soil explained 70 and 24.4% of the variation, respectively (Figure 6). There was a wide distance between the treatment and the control, indicating the differences in soil bacterial community functionality with or without the application of MWP-1.

#### 3.2.4 Effects of MWP-1 on soil nitrogen and phosphorus cycling genes

**Figure 7:**
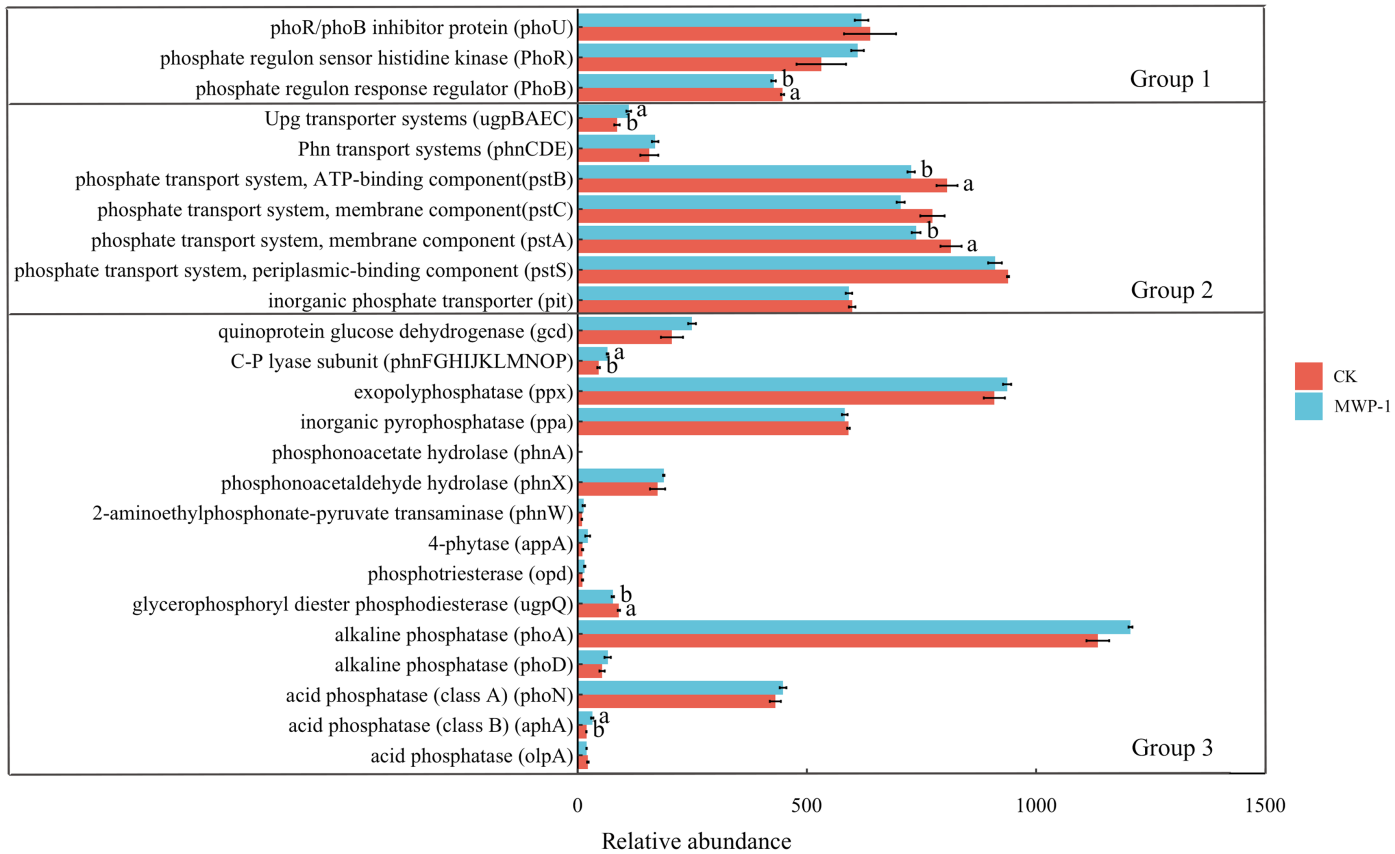
Relative abundance of major functional genes involved in phosphorus cycling. Group 1: Genes coding for phosphorus starvation response regulation; Group 2: Genes coding for phosphorus uptake and transport; Group 3: Genes coding for inorganic phosphorus solubilization and organic phosphorus mineralization. The relative abundance of the ugp-transporter system is calculated as the average abundance of genes *ugpB*, *ugpA*, *ugpE*, and *ugpC*. The relative abundance of the phn transporter system is calculated as the average abundance of genes *phnC*, *phnE*, and *phnD*. The relative abundance of the pst transporter system is calculated as the average abundance of genes *pstB*, *pstC*, *pstA*, and *pstS*. The relative abundance of the C- P lyase subunit is calculated as the average abundance of genes *phnF*, *phnG*, *phnH*, *phnI*, *phnJ*, *phnK*, *phnL*, *phnM*, *phnN*, *phnO*, and *phnP*. Different lowercase letters represent significant effects of MWP-1 application on the relative abundance of genes involved in phosphorus conversion (*p <* 0.05).

The genes involved in phosphorus conversion in soil phosphorus cycling can be classified into three categories by their functional roles: those involved in regulating microbial phosphorus starvation response, those involved in microbial inorganic phosphorus dissolution and organic phosphorus mineralization, and those involved in microbial phosphorus uptake and transport (Chen et al., 2024). As shown in Figure 7, the application of MWP-1 significantly reduced the relative abundance of *phoB* genes in the genome involved in regulating microbial phosphorus starvation response (*p <* 0.05), and significantly increased the relative abundance of the upg transport system in phosphorus uptake and transport (*p <* 0.05). The relative abundance of *pstB* and *pstA* genes significantly increased (*p <* 0.05). While the relative abundance of *upgQ* genes involved in inorganic phosphorus dissolution and organic phosphorus mineralization significantly decreased (*p <* 0.05), and the relative abundance of C-P system increased. The relative abundance of *aphA* genes significantly increased (*p <* 0.05).

**Figure 8:**
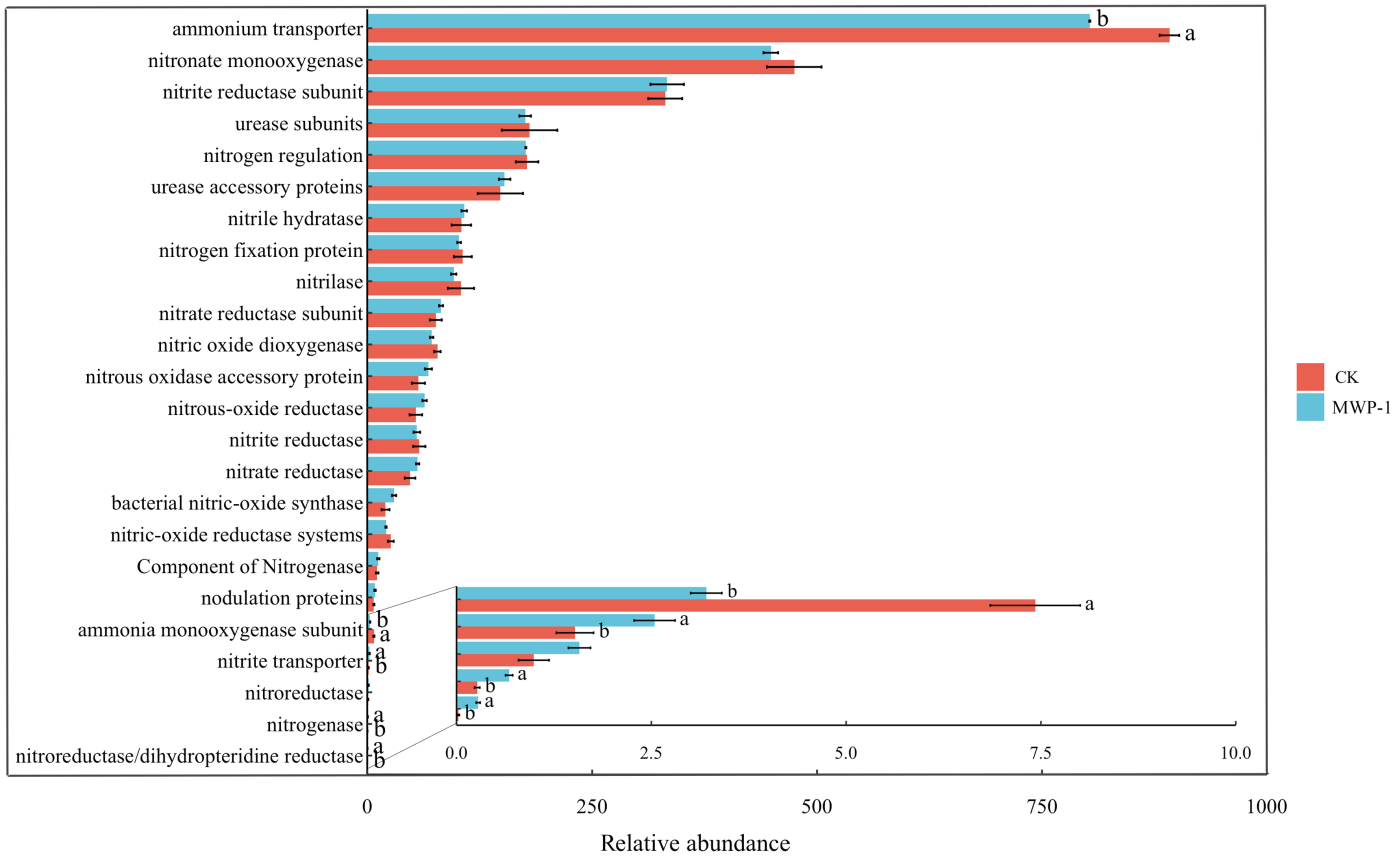
Relative abundance of major functional genes involved in nitrogen cycling. Different lowercase letters represent significant effects of MWP-1 application on the relative abundance of genes involved in nitrogen cycling (*p <* 0.05).

Most of the functional genes involved in nitrogen cycling, except for those involved in ammonia transporter, ammonia monooxygenase subunit, nitrate transporter, nitroeductaset, nitrogenase nitroreductase/dihydropteridine reductase, did not show any significant changes (Figure 8). The relative abundance of ammonia transporter and ammonia monooxygenase subunits in the genes regulating nitrogen cycling was significantly reduced (*p <* 0.05). The relative abundance of nitrite transporter, nitrogenase, and nitroreductase/dihydropteridinereductas significantly increased (*p <* 0.05).

### 3.3 Effects of MWP-1 on soil fungal communities

#### 3.3.1 Number of sequences

**Figure 9:**
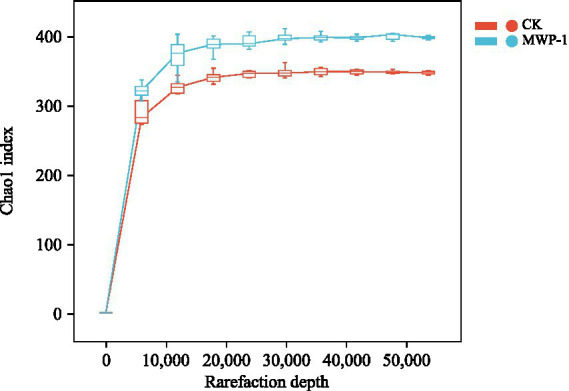
Sparse curve of sequencing sample dilution curve based on the Chao1 index of the fungal community.

On average, 84,751 valid sequences were identified per sample in the soil fungal community, and a total of 81,579 sequences remained after the removal of those ASVs with a total of 1 sequence. The dilution curves of the samples tend to flatten, indicating that the sequencing data were close to saturation, and the sequencing depth was reasonable (Figure 9).

#### 3.3.2 Soil fungal community composition

**Figure 10:**
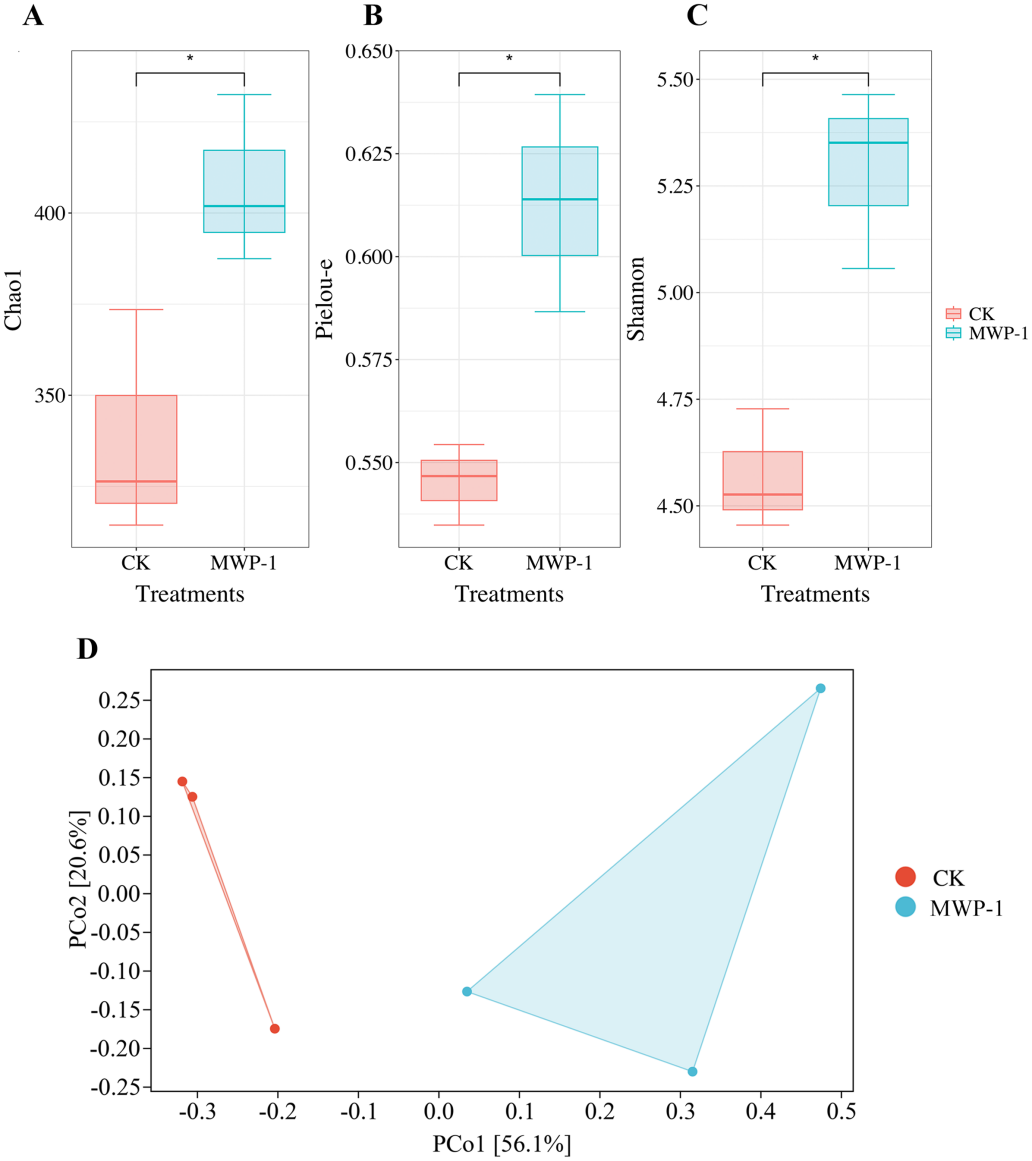
Analysis outcome of the alpha diversity indices: Chao1 index **(A)**, Pielou-e index **(B)**, Shannon index **(C)** and beta diversity **(D)** of the soil fungal community. *p < 0.05.

The Venn diagram showed a total of 228 ASVs obtained from the treatment and the control, of which 511 ASVs were unique to the control, and 573 ASVs unique to the treatment (Figure 10). The application of MWP-1 affected the number of ASVs in the soil fungi. The main microbial communities were observed as Ascomycota (51.60%), Basidiomycota (3.77%), and Glomeromycota (0.60%) at the phylum level in the treatment (Figure 10B). Some microbial communities were also detected, but at a lower relative abundance <0.1%, such as Mortierellomycota (0.02%), Chytridiomycota (0.052), Olpidiomycota (0.025%).

Heat map clustering showed that the MWP-1 treatment significantly enriched *Mycosphaerella, Coprinellus, Fusicolla, Radulidium, Rachicladosporium, Podospora, Gibberella, Schizochecium, Psathyrella, Serendipita, Curvularia, Allophaeosphaeria, Ceratobasidium, Elasticomyces,* and *Fontanospora* in comparison with the control (Figure 10C). However, the control had significantly enriched *Tricharina*, *Pseudaleuria*, *Preussia*, *Leptosphaeria*, *Cistella*, *Didymella*, *Thelebolus*, *Slopeiomyces*, *Paraphoma*, *Lecanicillium*, *Pseudogymnoascus*, *Tausonia*, *Tetracladium*, and *Coniochaeta*.

**Figure 11:**
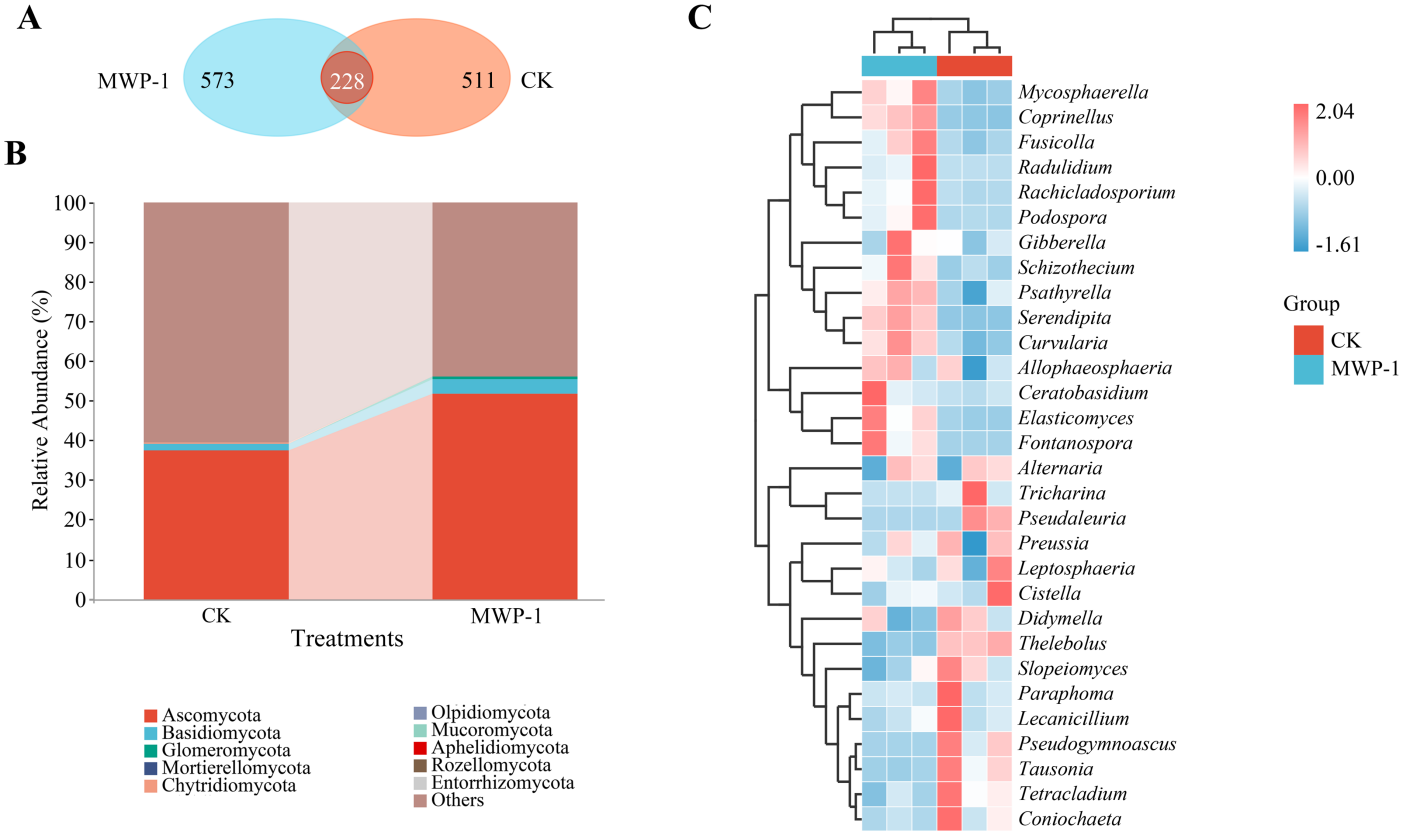
ASVs Wayne diagram **(A)**, horizontal community bar chart of bacterial phylum **(B)**, and thermal map of horizontal community composition of fungal genus **(C)** in the high-altitude mining area after the application of strain MWP-1.

The Chao1 index, Pielou-e index, and Shannon index of the fungal community significantly increased (*p <* 0.05) in the treatment (Figure 11), indicating that MWP-1 increased the diversity and evenness of the soil fungal communities in the alpine mining area. Analysis of fungal communities based on the Bray distance PCoA between samples showed that PCo1 and PCo2 contributed 56.1 and 20.6% to the total variation, respectively (Figure 11D). The soil fungal communities with or without the MWP-1 treatment were significantly separated on the PCo1 axis of PCoA, indicating that the MWP-1 affected the soil fungal community in the high-altitude mining area.

#### 3.3.3 Prediction of soil fungal community functionality

**Figure 12:**
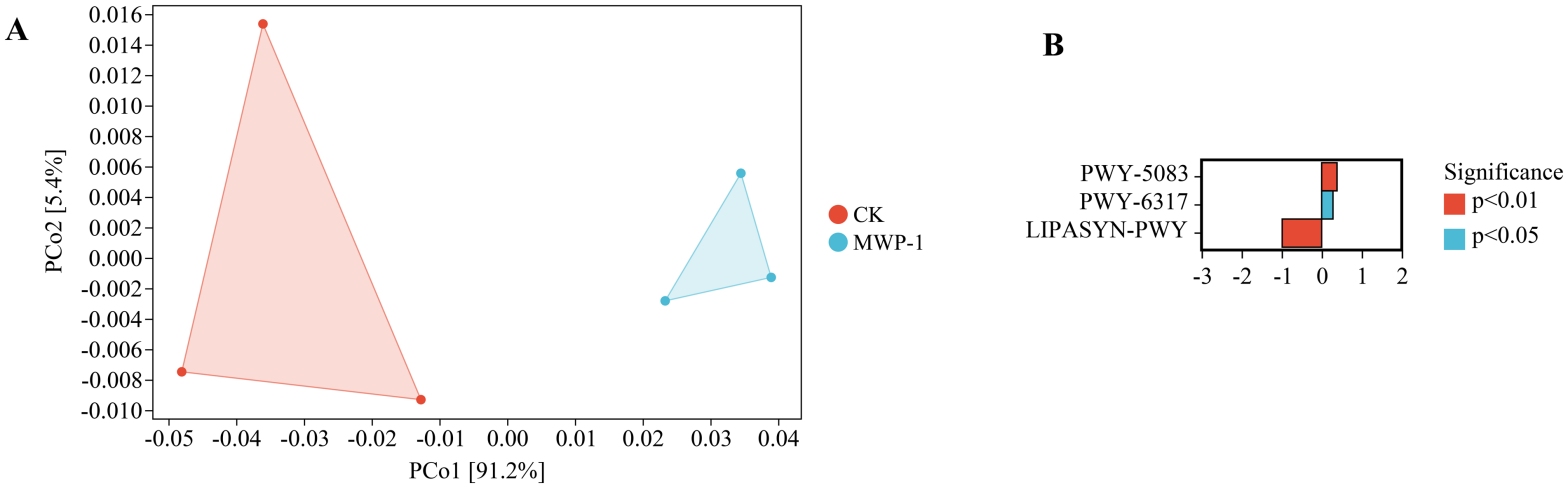
PICRUSt2 analysis of fungal community functional potential prediction. PCoA analysis of fungal community functional units **(A)** and statistical analysis of differences in metabolic pathways **(B)**.

The two dimensions of PCoA in soil explained 91.2 and 5.4% of the variation, respectively (Figure 12). The long distance between the treatment and control indicated that there were differences in fungal community functionality with or without the treatment of MWP-1. Analyses of metabolic pathways between groups with CK as the control and MWP-1 as the upregulated group showed that the treatment group had two upregulated metabolic pathways and one downregulated metabolic pathway, with highly significant differences (*p <* 0.01) between the two pathways, namely PWY-5083 pathway, NAD/NADH phosphorylation and dephosphorylation, and LIPASYN-PWY pathway, phosphorylation (Figure 12B). Therefore, MWP-1 had a significant impact on the metabolic functionality of soil fungal communities in the high-altitude mining area.

### 3.4 Relationship between microbial communities and environmental factors

**Figure 13:**
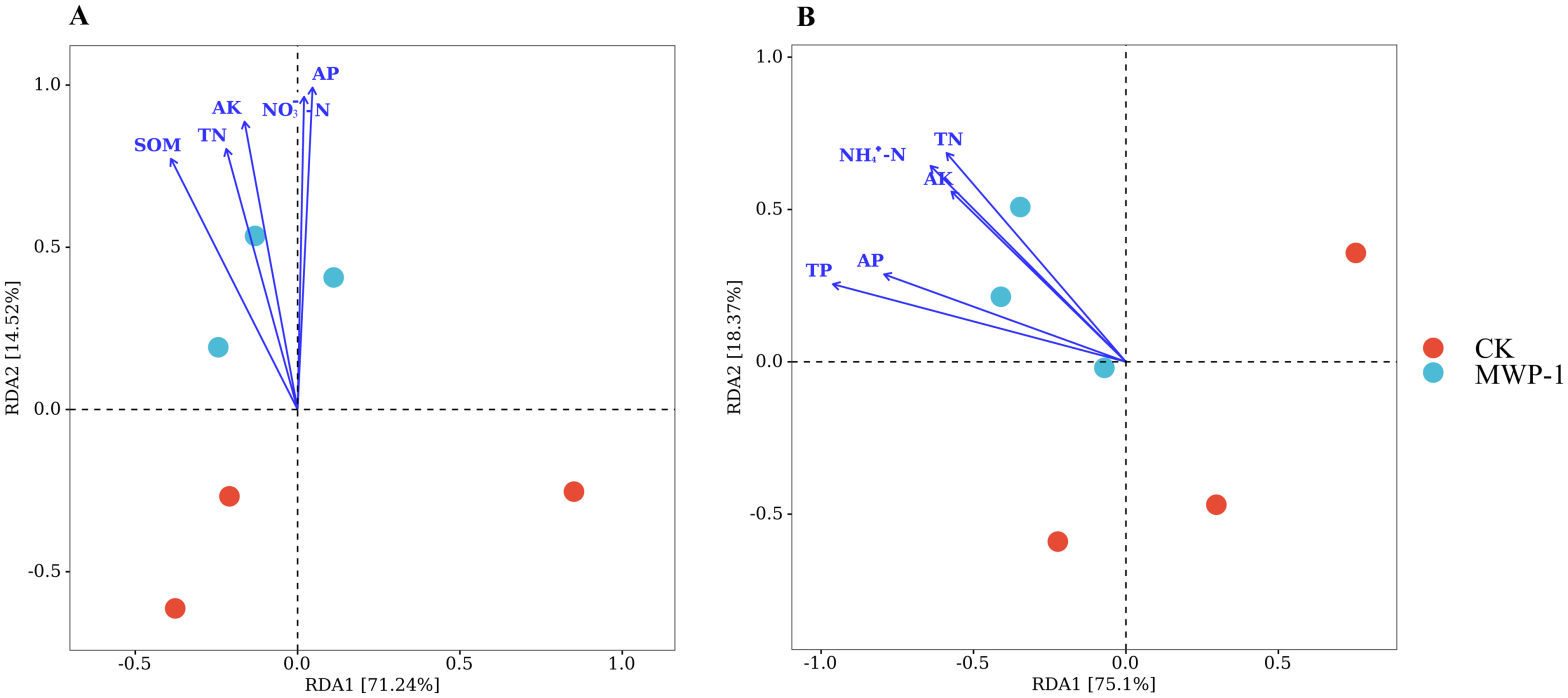
Redundancy analysis results of soil nutrients and the bacterial **(A)** and fungal **(B)** communities.

The redundancy analysis (RDA) of soil nutrients and soil microbial communities showed that the former had a significant impact on the latter. RDA1 and RDA2 explained 71.24 and 14.52% of the variation in the bacterial community structure caused by soil nutrients, respectively (Figure 13A). Among them, AP had the strongest impact on the bacterial community, followed by nitrate nitrogen and AK. RDA1 and RDA2 explained 75.1 and 18.37% of the variation in the fungal community caused by soil nutrients, respectively (Figure 13B). The soil total phosphorus content had the strongest impact on the fungal community, followed by ammonia nitrogen and soil total nitrogen content. This indicated that the improved soil nutrient content can effectively promote changes in soil microbial community, and the influencing factors differed between the bacterial and fungal communities.

## 4 Discussion

### 4.1 Effects of MWP-1 on vegetation properties and soil nutrients

This study analyzed the effects of applying MWP-1 on plant growth, soil nutrients, and soil microbial communities, and conducted a feasibility analysis for its use as a restoration resource for degraded grasslands in high-altitude mining areas. And it was found that under the special climate and environmental conditions of high-altitude regions, MWP-1 can significantly increase plant community coverage, height, and aboveground biomass, indicating that the interactions between PSB and plant roots or microbial communities can usually promote key processes conducive to plant growth (Mamta et al., 2010), thereby accelerating the increase in vegetation coverage. The average height and aboveground biomass of plants in the treatment were significantly higher than those in the control (*p <* 0.05), indicating a stronger growth potential. This change suggests an acceleration in the material cycle and energy flow of the ecosystem (Fujii et al., 2018). MWP-1 can also increase the content of available phosphorus in the soil. Through its unique phosphorus dissolution mechanism, PSB converts insoluble phosphorus compounds in the soil into available phosphorus that can be absorbed and utilized by plants (Pan and Cai, 2023), effectively alleviates the problem of soil phosphorus deficiency in the high-altitude mining area, and provides necessary nutritional support for vegetation growth (Yu et al., 2022). The total phosphorus content is an important indicator of total phosphorus storage in the soil. The PSB activity promotes the activation and transformation of soil phosphorus. And vegetation growth enriches soil organic matter, indirectly increasing soil total phosphorus levels (Alikhani et al., 2023). MWP-1 not only promotes the activation of phosphorus, but also enhances soil nitrogen cycling by improving the soil microenvironment. Besides, phosphorus solubilizing bacteria have a certain remedial effect on heavy metal pollution in mining areas. Various detoxification strategies can be used to reduce heavy metal toxicity, including direct adsorption, extracellular reduction, intracellular accumulation (Guo et al., 2021), biomineralization, and enzyme production (Zheng et al., 2021). Utilize biological metabolism to reduce the concentration of toxic and harmful substances in polluted environments or render them harmless. It can effectively cope with heavy metal damage.

The sequencing results indicate that MWP-1 has not become a dominant species in the soil of the mining area. Previously, PSB has been shown to have difficulty in colonizing bacteria in soil through a single application (Liu et al., 2020). In order to better promote the growth of exogenous PSB, Bamdad et al. (2022) proposed to combine bacteria with such carriers as biochar, perlite, peat, and so on. However, further experiments are needed to verify this claim. In spite of its failure to become the dominant species in the high-altitude mining area, MWP-1, a phosphate solubilizing bacterium, significantly promoted plant growth and increased the soil available phosphorus content. This indicates that it can survive and carry out normal life activities, play an efficient role in phosphate solubilization, promote plant growth and development, and increase plant height and aboveground biomass and the content of soil available nutrients, because PSB can promote the local enrichment of beneficial microorganisms in plant roots or rhizosphere soil (Buch and Gupta, 2024). Therefore, MWP-1 alone is able to address the soil nutrient deficiency problem in poor soils. The exogenous PSB MWP-1 changed soil properties. In turn, the changes in soil nutrient conditions can affect microbial communities and plant growth (Figure 13), and may affect microbial communities succession through plant root exudates (Anderson et al., 2024). Thus, the PSB selected in this study from the alpine meadow can adapt to the environment and climate of the alpine mining area, and can grow normally and play a promoting role in other alpine regions. Since MWP-1 was sourced locally, it would not cause invasion of foreign species.

### 4.2 Effect of MWP-1 on the composition diversity and function of soil microbial communities

The soil microbial communities are closely related to soil ecology and stability (Hu et al., 2021). This study has found that the impact of MWP-1 on the bacterial communities was mainly reflected at the taxonomic level below the phylum only (Figure 4B). At the genus level, MWP-1 increased the relative abundance of functional bacteria such as *Solirubrobacter* and *Rokubacteriales* (Figure 4C), which had a positive impact on nutrient cycling in the soil and the absorption and utilization of nutrients by plants, thereby affecting plant growth and development, boosting plant yield, and enhancing the disease resistance of the community (Lan et al., 2022; Jiang et al., 2023; Yang et al., 2023). The composition of soil fungal communities varied at the phylum level (Figure 10B), with more Ascomycota and Basidiomycota in the treatment than in the control, due possibly to the higher sensitivity of fungi to environmental changes (Jumpponen and Brown, 2014). As an important driver of carbon and nitrogen cycling and plant interactions (Challacombe et al., 2019), and also the main decomposer of soil organic matter, Ascomycota has a significant impact on plant growth. The decomposition of soil organic matter requires a large amount of nitrogen, and its increased abundance may be attributed to the nitrogenase secreted by Pseudomonas, which can provide sufficient nitrogen for Ascomycota and increase its abundance (Hu et al., 2023). Soil microbial diversity is an important indicator of community characteristics and stability. Following the MWP-1 application, the alpha diversity of the microbial communities increased, and the community became more complex (Zhou and Fong, 2021). The significant separation of beta diversity may be caused the increase of soil nutrients and root exudates can promote microbial communities succession, with species replacement or turnover (Legendre et al., 2005), and species loss or nesting in microbial communities (Matthews et al., 2019).

MWP-1 affects soil phosphorus cycling. Microbial high affinity (pst) transporters containing genes encoding phosphorus absorption and transport systems can absorb inorganic phosphorus under low phosphorus conditions (Gebhard et al., 2014). These genes regulating phosphorus absorption and transport enable microorganisms to effectively utilize phosphorus and fix it in microbial biomass (Mclaughlin et al., 1988). This is consistent with the gene level regulation of soil microbial communities’ functions by PSB in low phosphorus conditions in the mining area. The analysis of metabolic pathway differences in fungal communities showed that compared to the control, the LIPASYN-PWY pathway of phospholipases in the fungal community function of treatment showed significant downregulation, which may be due to the main role of PSB in dissolving insoluble phosphorus in the soil and converting it into available phosphorus that can be absorbed and utilized by plants. When the content of available phosphorus in the soil increases, the absorption and utilization efficiency of phosphorus by plants increases (Wang et al., 1995), which may lead to a relative decrease in organic phosphorus compounds such as phospholipids in the soil (Cardoso et al., 2003). Phospholipase, as a key enzyme for degrading phospholipids, may decrease its activity and the strength of its functional metabolic pathways due to the reduction of its substrate (phospholipids) (Fernández et al., 2012). Phospholipase activity is higher in phosphorus deficient soils (Richardson and Simpson, 2011), which confirms the promoting effect of PSB on soil phosphorus cycling (Li et al., 2023).

The relative abundance changes of nitrogen cycle related genes indicate the internal transformation of cycling processes and functional groups (Zhang et al., 2021). The results of this study indicate that soil microbial communities treated with MWP-1 have more active nitrogen metabolism. The effect of applying MWP-1 on soil nitrogen content may be due to nitrogen fixation and nitrification (Ward and Jensen, 2014). After applying MWP-1, the relative abundance of genes encoding nitrogenase and nitrite transport increased. Nitrogenase is a complex metalloenzyme that can break the very stable triple bonds in nitrogen molecules and convert them into ammonia that can be directly utilized by organisms (Loveless et al., 1999). Nitrification is a key process in the nitrogen cycle, and involves the oxidation of ammonia to nitrite (Kaur-Bhambra et al., 2022). An increase in the relative abundance of genes encoding nitrite can increase the substrates for nitrification and promote soil nitrogen cycling. The relative abundance of genes encoding both ammonium transporter and ammonia monooxygenase subunit decreases. Ammonium transporter can effectively affect the conversion and utilization efficiency of nitrogen elements, and its weakened function helps to retain nitrogen elements in the soil and reduce nitrogen loss (Chutrakul et al., 2022). The ammonia monooxygenase subunit is a key enzyme in the process of nitrification. The known aerobic ammonia oxidizing bacteria and archaea activate ammonia by oxidizing ammonia to hydroxylamine through ammonia monooxygenase (Arp and Stein, 2003). The weakening of the ammonia monooxygenase subunit can reduce ammonia volatilization loss and improve soil microbial activity (Leininger et al., 2006), which has a positive significance for maintaining soil fertility. Previous studies have shown that the application of bacterial fertilizer instead of 30% nitrogen fertilizer in a high-altitude mining area increased total nitrogen content, thereby promoting plant growth (Artyszak and Gozdowski, 2021).

## 5 Conclusion

MWP-1 can significantly increase coverage and height of vegetation, the aboveground biomass by more than 40%, the available phosphorus by >50%, the nitrate nitrogen content by 10% and the organic matter content by 10% in the soil in the high-altitude mining area. Although unable to become a dominant soil bacterium, MWP-1 can affect the succession of the microbial communities, increase communities’ diversity, alter microbial communities’ functions, and promote the transformation of soil nitrogen and phosphorus cycling functions. These findings can provide an excellent bacterial resource for the development of microbial fertilizers and the theoretical guidance for sustainable restoration of high-altitude mining areas.

## Data Availability

The original contributions presented in the study are included in the article/Supplementary material, further inquiries can be directed to the corresponding author.

## Footnotes

^1^ https://metacyc.org/

^2^ https://www.kegg.jp/
