# Supplementary material for: Impacts of phosphate-solubilizing bacterium strain MWP-1 on vegetation growth, soil characteristics, and microbial communities in the Muli coal mining area, China
Source: Front Microbiol. 2024 Dec 5;15:1500070. doi: 10.3389/fmicb.2024.1500070 (PMC11655473; doi:10.3389/fmicb.2024.1500070)
Supplement: Supplementary file 1 [file Table_1.docx]

The impact of phosphate solubilizing bacterium strain MWP-1 on soil characteristics and microbial community in the Muli coal mining area, China

**Table S1.** The KO number, KEGG descriptions, and classification of the genes responsible for microbial nitrogen metabolism and regulatory or transport.

| KO number | Classification | description |
| --- | --- | --- |
| K00360 | Nitrate reductase | *nasB;* assimilatory nitrate reductase electron transfer subunit |
| K00370 | Nitrate reductase subunit | *narG, narZ, nxrA*; nitrate reductase / nitrite oxidoreductase, alpha subunit |
| K00371 | Nitrate reductase subunit | *narH, narY, nxrB*; nitrate reductase / nitrite oxidoreductase, beta subunit |
| K00373 | Nitrate reductase subunit | *narJ, narW*; nitrate reductase molybdenum cofactor assembly chaperone NarJ/NarW |
| K00374 | Nitrate reductase subunit | *narI, narV*; nitrate reductase gamma subunit |
| K00372 | Nitrate reductase subunit | *nasA*; assimilatory nitrate reductase catalytic subunit |
| K05916 | Nitric oxide dioxygenase | *hmp*, YHB1; nitric oxide dioxygenase |
| K12265 | Nitric-oxide reductase systems | *norW*; nitric oxide reductase FlRd-NAD(+) reductase |
| K04747 | Nitric-oxide reductase systems | *norF*; nitric oxide reductase NorF protein |
| K04748 | Nitric-oxide reductase systems | *norQ*; nitric oxide reductase NorQ protein |
| K04561 | Nitric-oxide reductase systems | *norB*; nitric oxide reductase subunit B |
| K02448 | Nitric-oxide reductase systems | *norD*; nitric oxide reductase NorD protein |
| K02164 | Nitric-oxide reductase systems | *norE*; nitric oxide reductase NorE protein |
| K02305 | Nitric-oxide reductase systems | *norC*; nitric oxide reductase subunit C |
| K00491 | Bacterial nitric-oxide synthase | *nos;* nitric-oxide synthase, bacterial |
| K01501 | Nitrilase | E3.5.5.1; nitrilase |
| K01721 | Nitrile hydratase | *nthA*; nitrile hydratase subunit alpha |
| K00362 | Nitrite reductase subunit | *nirB*; nitrite reductase (NADH) large subunit |
| K00363 | Nitrite reductase subunit | *nirD*; nitrite reductase (NADH) small subunit |
| K00368 | Nitrite reductase | *nirK*; nitrite reductase (NO-forming) |
| K02598 | Nitrite transporter | *nirC*; nitrite transporter |
| K02585 | Nitrogen fixation protein | *nifB*; nitrogen fixation protein NifB |
| K02593 | Nitrogen fixation protein | *nifT*; nitrogen fixation protein NifT |
| K04488 | Nitrogen fixation protein | *iscU, nifU*; nitrogen fixation protein NifU and related proteins |
| K02595 | Nitrogen fixation protein | *nifW*; nitrogenase-stabilizing/protective protein |
| K02596 | Nitrogen fixation protein | *nifX*; nitrogen fixation protein NifX |
| K02597 | Nitrogen fixation protein | *nifZ*; nitrogen fixation protein NifZ |
| K10851 | Nitrogen regulation | *nreA*; nitrogen regulatory protein A |
| K02589 | Nitrogen regulation | *nifHD1, nifI1*; nitrogen regulatory protein PII 1 |
| K04751 | Nitrogen regulation | *glnB*; nitrogen regulatory protein P-II 1 |
| K02590 | Nitrogen regulation | *nifHD2, nifI2*; nitrogen regulatory protein PII 2 |
| K04752 | Nitrogen regulation | *glnK*; nitrogen regulatory protein P-II 2 |
| K07708 | Nitrogen regulation | *glnL, ntrB*; two-component system, NtrC family, nitrogen regulation sensor histidine kinase GlnL |
| K07712 | Nitrogen regulation | *glnG, ntrC*; two-component system, NtrC family, nitrogen regulation response regulator GlnG |
| K13598 | Nitrogen regulation | *ntrY*; two-component system, NtrC family, nitrogen regulation sensor histidine kinase NtrY |
| K13599 | Nitrogen regulation | *ntrX*; two-component system, NtrC family, nitrogen regulation response regulator NtrX |
| K02806 | Nitrogen regulation | *PTS-Ntr-EIIA, ptsN*; PTS system, nitrogen regulatory IIA component |
| K00531 | Nitrogenase | *anfG*; nitrogenase delta subunit |
| K02588 | Component of Nitrogenase | *nifH*; nitrogenase iron protein NifH |
| K02587 | Component of Nitrogenase | *nifE*; nitrogenase molybdenum-cofactor synthesis protein NifE |
| K02586 | Component of Nitrogenase | *nifD*; nitrogenase molybdenum-iron protein alpha chain |
| K02591 | Component of Nitrogenase | *nifK*; nitrogenase molybdenum-iron protein beta chain |
| K02592 | Component of Nitrogenase | *nifN*; nitrogenase molybdenum-iron protein NifN |
| K00459 | Nitronate monooxygenase | *ncd2*, npd; nitronate monooxygenase |
| K10679 | Nitroreductase/dihydropteridine reductase | *nfnB, nfsB*; nitroreductase / dihydropteridine reductase |
| K10678 | Nitroreductase | *nfsA*; nitroreductase |
| K07218 | Nitrous oxidase accessory protein | *nosD*; nitrous oxidase accessory protein |
| K00376 | Nitrous-oxide reductase | *nosZ*; nitrous-oxide reductase |
| K14658 | Nodulation proteins | *nodA*; nodulation protein A |
| K14660 | Nodulation proteins | *nodE*; nodulation protein E |
| K14661 | Nodulation proteins | *nodF*; nodulation protein F |
| K10944 | Ammonia monooxygenase subunit | *pmoA-amoA*; methane/ammonia monooxygenase subunit A |
| K10945 | Ammonia monooxygenase subunit | *pmoB-amoB*; methane/ammonia monooxygenase subunit B |
| K10946 | Ammonia monooxygenase subunit | *pmoC-amoC*; methane/ammonia monooxygenase subunit C |
| K03320 | Ammonium transporter | *amt*, AMT, MEP; ammonium transporter, Amt family |
| K03189 | Urease accessory proteins | *ureG*; urease accessory protein |
| K03188 | Urease accessory proteins | *ureF*; urease accessory protein |
| K03190 | Urease accessory proteins | *ureD, ureH*; urease accessory protein |
| K03187 | Urease accessory proteins | *ureE*; urease accessory protein |
| K03192 | Urease accessory proteins | *ureJ*; urease accessory protein |
| K01428 | Urease subunits | *ureC*; urease subunit alpha |
| K01430 | Urease subunits | *ureA*; urease subunit gamma |
| K01429 | Urease subunits | *ureB*; urease subunit beta |

**Table S2.** The KO number, KEGG descriptions, and classification of the genes responsible for microbial phosphorus transformation

| KO number | Classification | description |
| --- | --- | --- |
| K02039 | Genes involved in P-starvation response regulation | *phoU*; phosphate transport system protein |
| K07636 | Genes involved in P-starvation response regulation | *phoR*; two-component system, OmpR family, phosphate regulon sensor histidine kinase PhoR |
| K07657 | Genes involved in P-starvation response regulation | *phoB*; two-component system, OmpR family, phosphate regulon response regulator PhoB |
| K05813 | Genes involved in P-uptake and transport system | *ugpB*; sn-glycerol 3-phosphate transport system substrate-binding protein |
| K05814 | Genes involved in P-uptake and transport system | *ugpA*; sn-glycerol 3-phosphate transport system permease protein |
| K05815 | Genes involved in P-uptake and transport system | *ugpE*; sn-glycerol 3-phosphate transport system permease protein |
| K05816 | Genes involved in P-uptake and transport system | *ugpC*; sn-glycerol 3-phosphate transport system ATP-binding protein |
| K02041 | Genes involved in P-uptake and transport system | *phnC*; phosphonate transport system ATP-binding protein |
| K02042 | Genes involved in P-uptake and transport system | *phnE*; phosphonate transport system permease protein |
| K02044 | Genes involved in P-uptake and transport system | *phnD*; phosphonate transport system substrate-binding protein |
| K02036 | Genes involved in P-uptake and transport system | *pstB*; phosphate transport system ATP-binding protein |
| K02037 | Genes involved in P-uptake and transport system | *pstC*; phosphate transport system permease protein |
| K02038 | Genes involved in P-uptake and transport system | *pstA*; phosphate transport system permease protein |
| K02040 | Genes involved in P-uptake and transport system | *pstS*; phosphate transport system substrate-binding protein |
| K03306 | Genes involved in P-uptake and transport system | *TC.PIT*; inorganic phosphate transporter, PiT family |
| K00117 | Genes involved in inorganic P-solubilization and organic P-mineralization | *gcd*; quinoprotein glucose dehydrogenase |
| K05774 | Genes involved in inorganic P-solubilization and organic P-mineralization | *phnN*; ribose 1,5-bisphosphokinase |
| K05780 | Genes involved in inorganic P-solubilization and organic P-mineralization | *phnL*; alpha-D-ribose 1-methylphosphonate 5-triphosphate synthase subunit PhnL |
| K05781 | Genes involved in inorganic P-solubilization and organic P-mineralization | *phnK*; putative phosphonate transport system ATP-binding protein |
| K09994 | Genes involved in inorganic P-solubilization and organic P-mineralization | *phnO*; aminoalkylphosphonate N-acetyltransferase |
| K06162 | Genes involved in inorganic P-solubilization and organic P-mineralization | *phnM*; alpha-D-ribose 1-methylphosphonate 5-triphosphate diphosphatase |
| K06163 | Genes involved in inorganic P-solubilization and organic P-mineralization | *phnJ*; alpha-D-ribose 1-methylphosphonate 5-phosphate C-P lyase |
| K06164 | Genes involved in inorganic P-solubilization and organic P-mineralization | *phnI*; alpha-D-ribose 1-methylphosphonate 5-triphosphate synthase subunit PhnI |
| K06165 | Genes involved in inorganic P-solubilization and organic P-mineralization | *phnH*; alpha-D-ribose 1-methylphosphonate 5-triphosphate synthase subunit PhnH |
| K06166 | Genes involved in inorganic P-solubilization and organic P-mineralization | *phnG*; alpha-D-ribose 1-methylphosphonate 5-triphosphate synthase subunit PhnG |
| K06167 | Genes involved in inorganic P-solubilization and organic P-mineralization | *phnP*; phosphoribosyl 1,2-cyclic phosphate phosphodiesterase |
| K02043 | Genes involved in inorganic P-solubilization and organic P-mineralization | *phnF*; GntR family transcriptional regulator, phosphonate transport system regulatory protein |
| K01524 | Genes involved in inorganic P-solubilization and organic P-mineralization | *ppx-gppA*; exopolyphosphatase / guanosine-5'-triphosphate,3'-diphosphate pyrophosphatase |
| K01507 | Genes involved in inorganic P-solubilization and organic P-mineralization | *ppa*; inorganic pyrophosphatase |
| K06193 | Genes involved in inorganic P-solubilization and organic P-mineralization | *aphA*; acid phosphatase (class B) |
| K05306 | Genes involved in inorganic P-solubilization and organic P-mineralization | *phnA*; protein PhnA |
| K03430 | Genes involved in inorganic P-solubilization and organic P-mineralization | *phnX*; phosphonoacetaldehyde hydrolase |
| K01093 | Genes involved in inorganic P-solubilization and organic P-mineralization | *phnW*; 2-aminoethylphosphonate-pyruvate transaminase |
| K07048 | Genes involved in inorganic P-solubilization and organic P-mineralization | *appA*; 4-phytase / acid phosphatase |
| K01126 | Genes involved in inorganic P-solubilization and organic P-mineralization | *PTER*, php; phosphotriesterase-related protein |
| K01077 | Genes involved in inorganic P-solubilization and organic P-mineralization | *glpQ, ugpQ*; glycerophosphoryl diester phosphodiesterase |
| K01113 | Genes involved in inorganic P-solubilization and organic P-mineralization | *phoA, phoB*; alkaline phosphatase |
| K09474 | Genes involved in inorganic P-solubilization and organic P-mineralization | *phoD*; alkaline phosphatase D |
| K03788 | Genes involved in inorganic P-solubilization and organic P-mineralization | *phoN*; acid phosphatase (class A) |
| K01078 | Genes involved in inorganic P-solubilization and organic P-mineralization | *PHO*; acid phosphatase |
